# Diabetes, Adiposity, and Functional Phenotypes in HFpEF: A Systematic Review of Recent Treatment-Response Evidence

**DOI:** 10.7759/cureus.114404

**Published:** 2026-08-12

**Authors:** Mulusew Goshe, Sarah Singh, Rabia Zameer, Jawad Ali, Neha Kumari, Mehta Das

**Affiliations:** 1 Family Medicine, MultiCare Health System, Tacoma, USA; 2 Psychiatry and Behavioral Sciences, Vita-Salute San Raffaele University, Milan, ITA; 3 Internal Medicine, Larkin Community Hospital, South Miami, USA; 4 Internal Medicine, Shaheed Mohtarma Benazir Bhutto Medical University, Larkana, PAK; 5 Internal Medicine, Ghulam Muhammad Mahar Medical College, Sukkur, PAK; 6 Internal Medicine, Jinnah Hospital, Lahore, PAK

**Keywords:** cardiometabolic phenotype, heart failure with preserved ejection fraction, obesity, treatment response, type 2 diabetes

## Abstract

Heart failure with preserved ejection fraction (HFpEF) is increasingly understood as a heterogeneous syndrome in which cardiometabolic and functional phenotypes may influence treatment response beyond ejection fraction alone. This systematic review synthesized recent clinical and trial-derived evidence published from June 2025 to May 2026 evaluating cardiometabolic phenotypes and therapeutic response in adults with HFpEF or closely related heart failure with mildly reduced or preserved ejection fraction populations. PubMed/MEDLINE, Scopus, and Web of Science were searched according to the Preferred Reporting Items for Systematic Reviews and Meta-Analyses 2020 principles using terms related to HFpEF, diabetes, obesity, adiposity, frailty, exercise function, sodium-glucose cotransporter 2 inhibitors, incretin-based therapies, and treatment outcomes. Six studies met the eligibility criteria, including randomized trials, prespecified secondary analyses, pooled participant-level analyses, stratification-based analyses, and randomized crossover trial evidence. The included studies evaluated tirzepatide, semaglutide, dapagliflozin, and dapagliflozin plus spironolactone across phenotypes including type 2 diabetes, body mass index (BMI), central adiposity, achieved weight loss, epicardial or paracardiac fat, exercise limitation, frailty, and renal-electrolyte profile. Overall, contemporary cardiometabolic therapies were associated with improvements in heart failure outcomes, patient-reported health status, exercise capacity, body weight, natriuretic peptides, adiposity measures, cardiac remodeling markers, and frailty burden. Diabetes did not appear to uniformly attenuate heart failure benefit despite less weight loss, while adiposity distribution, functional limitation, frailty, and local cardiac fat may represent clinically relevant, hypothesis-generating domains for interpreting treatment response. Sodium-glucose cotransporter 2 inhibitor-based therapy also showed potential cardiometabolic, renal, hemodynamic, and structural effects, although combination therapy required careful renal and potassium monitoring. These findings suggest the potential value of a phenotype-informed framework for evaluating cardiometabolic treatment response in HFpEF; however, they are insufficient to support phenotype-guided treatment selection because the evidence was derived predominantly from secondary, pooled, or subgroup analyses rather than prospective phenotype-guided trials. Prospective studies are needed to determine whether integrating diabetes status, adiposity distribution, exercise capacity, frailty, renal profile, and imaging-based fat depots can improve treatment selection beyond ejection fraction and body mass index alone.

## Introduction and background

Heart failure with preserved ejection fraction (HFpEF) is increasingly recognized as a heterogeneous clinical syndrome rather than a single disease entity. Although left ventricular ejection fraction remains central to classification, it does not adequately explain the marked variation in symptoms, exercise capacity, congestion, metabolic burden, comorbidity profile, or treatment response observed across patients [1]. This heterogeneity has encouraged a shift toward phenotype-based interpretation. In this review, a phenotype refers to a clinically identifiable pattern defined by cardiometabolic, functional, renal, or imaging-related characteristics that may be associated with differences in disease expression or therapeutic response. This approach is particularly relevant in patients with obesity, type 2 diabetes, systemic inflammation, renal dysfunction, frailty, and impaired functional reserve. These features are not merely coexisting conditions; they may influence HFpEF pathophysiology through interconnected hemodynamic, metabolic, inflammatory, skeletal muscle, vascular, and myocardial mechanisms [2].

Diabetes and obesity are especially important in contemporary HFpEF because they frequently coexist and may define a cardiometabolic phenotype characterized by greater symptom burden, impaired exercise tolerance, altered body composition, ectopic adiposity, renal vulnerability, and systemic metabolic dysfunction [3]. However, the clinical meaning of these phenotypes remains complex. For example, diabetes may be associated with less weight loss after incretin-based therapy, but this does not necessarily indicate attenuated heart failure benefit. Similarly, body mass index (BMI) provides a useful estimate of global adiposity but may not fully capture central adiposity, epicardial or paracardiac fat, inflammatory burden, or the functional consequences of obesity-related HFpEF. These distinctions are clinically relevant because treatment response may be reflected through symptoms, six-minute walk distance (6MWD), natriuretic peptides, body composition, cardiac remodeling, renal indices, or frailty burden rather than weight change alone [4].

Recent therapeutic developments have further strengthened the need to examine HFpEF through a cardiometabolic lens. Sodium-glucose cotransporter 2 (SGLT2) inhibitors, incretin-based therapies, and combination strategies involving mineralocorticoid receptor antagonism have generated evidence across clinical, functional, biomarker, structural, and safety domains [5]. Given the rapid expansion of this evidence, the review was intentionally restricted to the most recent 12-month period to provide a focused update on emerging phenotype-related findings that may not have been captured in earlier, broader reviews. At the same time, many recent data come from secondary analyses, stratification-based analyses, pooled participant-level analyses, or phenotype-focused trial analyses rather than trials primarily designed to test phenotype-guided treatment selection. This creates an important interpretive gap: current evidence suggests that cardiometabolic and functional phenotypes may shape treatment response, but the extent to which these markers should guide therapeutic selection in routine HFpEF care remains uncertain [6].

This systematic review aims to synthesize recent clinical and trial-derived evidence evaluating cardiometabolic phenotypes and treatment response in adults with HFpEF or closely related heart failure with mildly reduced or preserved ejection fraction populations. Specifically, it examines whether diabetes, obesity severity, central adiposity, epicardial or paracardiac fat, exercise limitation, frailty, and renal-electrolyte profile are associated with differences in response to contemporary therapies, including tirzepatide, semaglutide, dapagliflozin, and dapagliflozin-based combination therapy. By integrating clinical outcomes, patient-reported health status, functional capacity, biomarkers, adiposity measures, cardiac remodeling, and safety outcomes, this review seeks to clarify whether the most recent evidence supports a phenotype-informed framework for interpreting cardiometabolic treatment response in HFpEF.

## Review

Materials and methods

Study Design and Reporting Framework

This systematic review was designed to evaluate recent clinical evidence on cardiometabolic phenotypes and treatment response in HFpEF. The review was conducted in accordance with the Preferred Reporting Items for Systematic Reviews and Meta-Analyses (PRISMA) 2020 statement [7]. The research question was structured using the PICO framework (Patient/Population, Intervention, Comparator/Control, and Outcome(s)) [8]. The review protocol was not prospectively registered in PROSPERO because the study was conceived as a focused, time-limited synthesis of rapidly emerging evidence published within a predefined 12-month period. Nevertheless, the research question, eligibility criteria, phenotype domains, interventions, and outcomes were defined before study selection. The absence of prospective protocol registration is acknowledged as a methodological limitation because it reduces external verification of the prespecified methods. The population of interest comprised adults with HFpEF, obesity-related HFpEF, or closely related mildly reduced/preserved ejection fraction heart failure populations when HFpEF-specific relevance was clear. The exposure or phenotype domain included type 2 diabetes, obesity, central adiposity, epicardial or paracardiac adipose tissue, frailty, exercise limitation, renal profile, and other cardiometabolic modifiers. The interventions of interest included contemporary cardiometabolic therapies, particularly incretin-based therapies and SGLT2 inhibitor-based strategies. Comparators included placebo, active treatment comparators, or phenotype-based subgroups within randomized trial populations. Outcomes of interest included cardiovascular death, worsening heart failure, heart failure hospitalization, patient-reported health status, Clinical Summary Score of the Kansas City Cardiomyopathy Questionnaire (KCCQ-CSS), 6MWD, body weight, waist measures, natriuretic peptides, inflammatory markers, renal indices, potassium, cardiac remodeling measures, epicardial or paracardiac fat, and frailty burden.

Search Strategy and Data Sources

A systematic literature search was performed in PubMed/MEDLINE, Scopus, and Web of Science to identify relevant studies published from June 1, 2025, to May 31, 2026. The 12-month interval was selected a priori because the review was designed as a focused contemporary update rather than an exhaustive synthesis of the entire historical HFpEF literature. This period coincided with the emergence of several trial-derived and phenotype-focused analyses of incretin-based therapies, SGLT2 inhibitor strategies, adiposity distribution, frailty, exercise limitation, and related cardiometabolic domains. Restricting the search to this interval enabled concentrated evaluation of evidence generated after broader reviews and major HFpEF trials had established the general therapeutic efficacy of these drug classes. The objective was therefore to examine how recently reported phenotypic characteristics might modify or contextualize treatment response, rather than to reassess the overall efficacy of established HFpEF therapies. Nevertheless, the restricted timeframe may have excluded relevant earlier or foundational studies and was considered when interpreting the completeness and generalizability of the evidence. The final primary evidence base included recent randomized trials, prespecified secondary analyses, pooled participant-level analyses, stratification-based analyses, and randomized crossover trial evidence that met the predefined eligibility criteria. The search strategy combined controlled vocabulary and free-text terms related to HFpEF, diabetes, cardiometabolic phenotype, adiposity, frailty, exercise function, and contemporary therapies. Boolean operators were used to combine search concepts. A representative PubMed search string included: (“heart failure with preserved ejection fraction” OR “HFpEF” OR “Heart Failure, Diastolic”) AND (“diabetes” OR “type 2 diabetes” OR “T2DM” OR “obesity” OR “adiposity” OR “frailty” OR “epicardial adipose tissue” OR “exercise function”) AND (“tirzepatide” OR “semaglutide” OR “dapagliflozin” OR “SGLT2 inhibitor” OR “spironolactone”) AND (“treatment response” OR “clinical outcomes” OR “KCCQ” OR “6-minute walk distance” OR “NT-proBNP” OR “cardiovascular death” OR “worsening heart failure”). Search terms were adapted for each database while maintaining the same conceptual structure. Reference lists of relevant articles were also reviewed to identify additional eligible studies.

Eligibility Criteria

Studies were eligible for inclusion if they evaluated adults with HFpEF, obesity-related HFpEF, or HFmrEF/HFpEF populations and examined the association between cardiometabolic phenotypes and treatment response. Eligible phenotypes included type 2 diabetes, BMI, central adiposity, achieved weight loss, waist-height ratio, epicardial or paracardiac adipose tissue, baseline exercise limitation, frailty status, renal profile, or related cardiometabolic modifiers. Eligible interventions included incretin-based therapies such as tirzepatide or semaglutide, SGLT2 inhibitor therapy, such as dapagliflozin, or SGLT2 inhibitor-based combination therapy with mineralocorticoid receptor antagonism. Randomized controlled trials, prespecified secondary analyses, participant-level pooled analyses, stratification-based analyses, blinded-endpoint crossover trials, and pooled analyses of randomized placebo-controlled trials were considered eligible when they provided clinically relevant data on treatment response, functional outcomes, biomarker change, adiposity remodeling, or safety. Studies were excluded if they were narrative reviews, editorials, case reports, animal studies, nonclinical mechanistic studies, or studies that did not include heart failure, preserved or mildly reduced/preserved ejection fraction, cardiometabolic phenotype assessment, or treatment-response outcomes relevant to the review question. No language restrictions were applied during database searching or study selection.

Study Selection

All records identified through database searching were imported into a reference management workflow, and duplicates were removed before screening. Two reviewers independently screened titles and abstracts for relevance. Full texts of potentially eligible studies were then assessed by the same two reviewers against the predefined eligibility criteria. Disagreements at either screening stage were resolved through discussion and, when needed, adjudication by a third reviewer. A formal measure of inter-reviewer agreement, such as Cohen’s kappa, was not calculated. The study selection process was documented according to PRISMA 2020 principles, including the number of records identified, screened, excluded, assessed in full text, and included in the final synthesis.

Data Extraction

Data were extracted using a structured extraction table developed before final synthesis. Extracted variables included first author, year of publication, study design, trial source or analysis type, sample size, heart failure population, phenotype or modifier assessed, intervention and comparator, follow-up duration, outcomes extracted, and key findings. Particular attention was given to whether treatment response differed by diabetes status, adiposity severity, central adiposity, exercise function, frailty status, epicardial or paracardiac fat, renal parameters, or safety profile. Outcomes were grouped into clinical outcomes, patient-reported outcomes, functional outcomes, biomarker outcomes, adiposity or remodeling outcomes, and safety outcomes. Data extraction was performed independently by two reviewers and cross-checked for accuracy. Any disagreements were resolved through discussion and consensus; if consensus could not be reached, a third reviewer adjudicated the discrepancy.

Risk-of-Bias Assessment

Risk of bias was assessed using the revised Cochrane Risk of Bias tool for randomized trials. Two reviewers independently performed the RoB 2 assessment for each included study. For the crossover study, the RoB 2 approach was applied with attention to design-specific issues relevant to crossover trials [9], including deviations from intended interventions, carryover considerations, endpoint assessment, and missing outcome data. Because several included studies were secondary, pooled, or subgroup analyses of randomized trial data, additional caution was applied when interpreting effect modification, subgroup findings, and exploratory associations. Each study was categorized as low risk of bias, some concerns, or high risk of bias. Disagreements between reviewers were resolved through discussion and consensus, with third-reviewer adjudication when required.

Data Synthesis

A narrative synthesis was performed because the included studies differed substantially in intervention type, phenotype assessed, outcome structure, follow-up duration, and analytic approach. Quantitative meta-analysis was not performed because the evidence base included heterogeneous analyses of tirzepatide, semaglutide, dapagliflozin, and dapagliflozin/spironolactone. Clinical heterogeneity arose from differences in heart failure populations, ejection-fraction eligibility criteria, diabetes status, obesity severity, frailty, exercise limitation, renal profile, and baseline risk. Methodological heterogeneity resulted from the inclusion of primary randomized trials, crossover evidence, prespecified secondary analyses, pooled participant-level analyses, and exploratory subgroup or stratification-based analyses. Outcomes also differed in their definitions, measurement instruments, assessment time points, and reported effect measures, spanning clinical events, KCCQ-CSS, 6MWD, body weight, waist measures, inflammatory markers, natriuretic peptides, renal indices, cardiac remodeling, epicardial adipose tissue, and frailty burden. These differences precluded identification of a sufficiently comparable group of studies for a statistically and clinically meaningful pooled estimate. Findings were therefore synthesized thematically around diabetes status, adiposity phenotype, exercise limitation, frailty, local adiposity remodeling, and SGLT2-based cardiometabolic optimization. This synthesis strategy was selected to preserve clinical interpretability and identify whether recent evidence supports a phenotype-informed framework for treatment-response assessment in HFpEF.

Results

Study Selection Process

**Figure 1:**
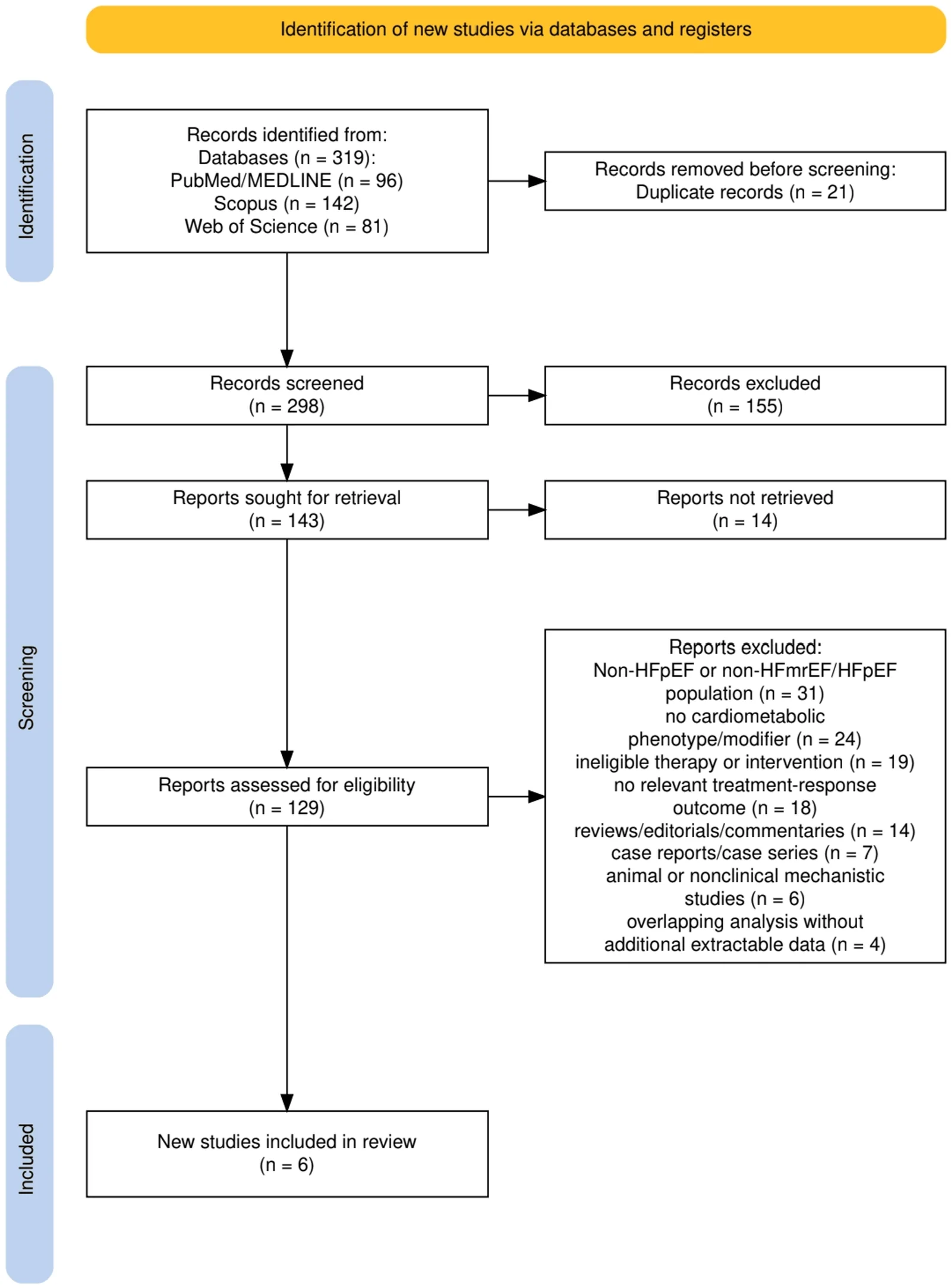
PRISMA flow diagram of the study identification, screening, eligibility assessment, and inclusion process. PRISMA: Preferred Reporting Items for Systematic Reviews and Meta-Analyses

Figure 1 summarizes the PRISMA 2020 study-selection process. The database search identified 319 records, including 96 from PubMed/MEDLINE, 142 from Scopus, and 81 from Web of Science. After removal of 21 duplicates, 298 records were screened, of which 155 were excluded at the title and abstract stage. Conference abstracts and preprints identified during screening were considered for relevance but were excluded from the final evidence base because only full-text, peer-reviewed studies providing sufficient methodological and outcome data were eligible. A total of 143 reports were sought for retrieval, with 14 not retrieved, leaving 129 reports for full-text eligibility assessment. Of these, 123 were excluded for predefined reasons, most commonly non-HFpEF or non-HFmrEF/HFpEF populations, absence of a relevant cardiometabolic phenotype or modifier, ineligible therapy or intervention, or lack of treatment-response outcomes. Ultimately, six studies met the eligibility criteria and were included in the final systematic review. All included studies were published within the predefined 12-month period from June 1, 2025, to May 31, 2026.

Characteristics of the Selected Studies

The characteristics of the included studies are summarized in Table 1. The final evidence base consisted of recent randomized and trial-derived studies evaluating cardiometabolic and functional phenotypes in HFpEF or closely related HFmrEF/HFpEF populations. The included studies examined incretin-based therapy with tirzepatide or semaglutide, SGLT2 inhibitor therapy with dapagliflozin, and dapagliflozin-based combination therapy with spironolactone. Across these studies, the main phenotypes or modifiers assessed included type 2 diabetes, obesity severity, central adiposity, achieved weight loss, epicardial or paracardiac adipose tissue, baseline exercise limitation, frailty status, and renal-electrolyte profile. The extracted outcomes included clinical events, patient-reported health status, functional capacity, biomarkers, adiposity and remodeling measures, and safety outcomes, allowing synthesis of treatment response across multiple clinically relevant domains.

**Table 1:** Summary of the included studies evaluating cardiometabolic phenotypes and treatment response in HFpEF. Note: Superscripts denote overlapping participant populations: a, analyses of the SUMMIT trial population (n = 731) [10,11]; b, analyses of the pooled STEP-HFpEF and STEP-HFpEF DM populations (n = 1,145) [13,15]. These publications were synthesized separately but were not treated as independent participant cohorts. HFpEF, heart failure with preserved ejection fraction; NYHA, New York Heart Association; BMI, body mass index; WHR, waist-height ratio; HF, heart failure; CV, cardiovascular; KCCQ-CSS, Kansas City Cardiomyopathy Questionnaire Clinical Summary Score; 6MWD, six-minute walk distance; CRP, C-reactive protein; BP, blood pressure; QoL, quality of life; LV, left ventricular; CMR, cardiac magnetic resonance; TNF, tumor necrosis factor; IL, interleukin; EAT, epicardial adipose tissue; HFrEF, heart failure with reduced ejection fraction; STEP-HFpEF, semaglutide treatment effect in people with obesity and heart failure with preserved ejection fraction; STEP-HFpEF DM, semaglutide treatment effect in people with obesity, heart failure with preserved ejection fraction, and diabetes mellitus; NT-proBNP, N-terminal pro-B-type natriuretic peptide; HFmrEF, heart failure with mildly reduced ejection fraction; EF, ejection fraction; eGFR, estimated glomerular filtration rate

Study	Design	Population	Phenotype / modifier assessed	Therapy / comparator	Outcomes extracted	Key findings
Borlaug et al., 2025 [10]^a^	Secondary analysis of randomized controlled trial	731 patients with obesity-related HFpEF, NYHA class II-IV, BMI ≥30 kg/m²; tirzepatide n=364, placebo n=367	Baseline obesity severity by BMI, central adiposity by waist-height ratio, achieved weight loss, and waist change	Tirzepatide vs. placebo	Cardiovascular death or worsening HF; KCCQ-CSS; 6MWD; CRP; body weight; waist circumference; systolic BP	Tirzepatide reduced worsening HF/CV death consistently across BMI and WHR groups. Higher baseline BMI was associated with greater improvement in 6MWD and greater reductions in body weight and systolic BP. Greater achieved weight loss was associated with larger improvements in 6MWD, KCCQ-CSS, CRP, and BP.
Packer et al., 2025 [11]^a^	Prespecified stratification-based analysis of randomized controlled trial	731 patients with HFpEF and obesity, BMI ≥30 kg/m²; randomized 1:1; median follow-up 104 weeks	Type 2 diabetes status; body-weight loss; paracardiac fat; LV mass	Tirzepatide up to 15 mg weekly vs. placebo	Cardiovascular death or worsening HF; KCCQ-CSS; 6MWD; QoL scores; NYHA class; body weight; LV mass; paracardiac fat; safety	Tirzepatide reduced cardiovascular death or worsening HF overall, with similar treatment effects in patients with and without diabetes. Functional, symptom, QoL, and NYHA improvements did not differ by diabetes status. Weight loss was smaller in patients with diabetes, but reductions in paracardiac fat and LV mass were similar across diabetes groups.
Alghamdi et al., 2025 [12]	Pooled analysis of two double-blind, placebo-controlled randomized trials	122 adults with type 2 diabetes and stage B/C HF; obesity common; HFpEF 51%, HFrEF 49%	Type 2 diabetes; obesity; epicardial adipose tissue; cardiac remodeling phenotype	Dapagliflozin 10 mg once daily vs. placebo for 12 months	Epicardial adipose tissue by CMR; BMI; LV mass; TNF, IL-1, IL-6, IL-10, and CRP	Dapagliflozin significantly reduced epicardial adipose tissue, BMI, and LV mass compared with placebo. EAT reduction occurred independently of BMI change. No parallel systemic inflammatory change was observed, suggesting a primarily local cardiometabolic mechanism.
Borlaug et al., 2025 [13]^b^	Prespecified secondary analysis of pooled randomized controlled trial data	1,145 patients with obesity-related HFpEF, including STEP-HFpEF and STEP-HFpEF DM populations	Baseline exercise function by 6MWD; adiposity; congestion; inflammation; achieved weight loss	Semaglutide vs. placebo	6MWD at 20 and 52 weeks; KCCQ-CSS; body weight; CRP; NT-proBNP; hierarchical composite including death, HF events, KCCQ-CSS, and 6MWD	Lower baseline 6MWD was associated with older age, worse KCCQ-CSS, higher BMI and waist circumference, greater CRP, higher NT-proBNP, and more diuretic use. Semaglutide improved 6MWD at 20 and 52 weeks, improved KCCQ-CSS, reduced body weight, CRP, and NT-proBNP, with consistent effects across baseline 6MWD groups.
Ferreira et al., 2025 [14]	Prospective randomized open-label, blinded-endpoint crossover trial	108 patients with HFpEF or mildly reduced/preserved EF; median age 76 years; 57% women; 45% had diabetes	HFpEF/HFmrEF cardiometabolic treatment phenotype; diabetes as baseline characteristic; renal and potassium safety profile	Dapagliflozin + spironolactone vs. dapagliflozin alone; each sequence for 12 weeks	NT-proBNP; ≥20% NT-proBNP reduction; systolic BP; urinary albumin-to-creatinine ratio; eGFR; serum potassium; hyperkalemia	Compared with dapagliflozin alone, dapagliflozin/spironolactone reduced log NT-proBNP and increased the odds of ≥20% NT-proBNP reduction. It also reduced systolic BP and albuminuria but caused greater eGFR decline, higher potassium, and more hyperkalemia.
Pandey et al., 2025 [15]^b^	Prespecified pooled participant-level analysis of randomized clinical trial data	1,145 patients with obesity-related HFpEF from the STEP-HFpEF program; semaglutide 2.4 mg vs. placebo for 52 weeks	Frailty status using a 34-variable cumulative deficit frailty index; baseline strata: nonfrail, more frail, most frail	Semaglutide 2.4 mg once weekly vs. placebo	KCCQ-CSS; body weight; frailty burden at follow-up; safety	Most participants were frail at baseline. Semaglutide produced similar weight loss across frailty strata, but symptom improvement varied by frailty status, with the greatest KCCQ-CSS improvement among the most frail patients. Semaglutide also reduced frailty burden at 52 weeks, increasing the odds of being nonfrail.

Risk-of-Bias Assessment

The risk-of-bias assessment is presented in Table 2. Overall, the included studies were supported by randomized trial designs or randomized trial-derived datasets, which strengthened the internal validity of the evidence base. The analysis by Packer et al. was judged to have low risk of bias because diabetes status was incorporated as a prespecified stratification variable, and the analysis retained the randomized treatment comparison [11]. The remaining studies were judged as having some concerns for study-specific reasons. In the SUMMIT (A Study of Tirzepatide in Participants With Heart Failure With Preserved Ejection Fraction and Obesity) secondary analysis by Borlaug et al., concerns arose from multiple phenotype-based comparisons and analyses relating outcomes to achieved weight loss, a post-randomization variable susceptible to residual confounding [10]. The pooled analysis by Alghamdi et al. combined data from two trials and included mixed HFpEF and HFrEF populations, raising concerns regarding population heterogeneity and indirectness for HFpEF-specific conclusions [12]. The pooled STEP-HFpEF analysis by Borlaug et al. evaluated treatment effects across baseline 6MWD strata and multiple secondary outcomes, creating some concern regarding multiplicity and selection of reported subgroup results [13]. The crossover trial by Ferreira et al. raised design-specific concerns related to its open-label treatment allocation, potential period or carryover effects, and short treatment periods, although blinded endpoint assessment reduced measurement-related bias [14]. The pooled analysis by Pandey et al. used a derived frailty index and examined treatment effects across multiple frailty strata, raising concerns related to subgroup construction, multiplicity, and exploratory effect-modification analyses [15]. No study was judged to be at high risk of bias; however, these limitations warrant cautious interpretation of the phenotype-specific findings.

**Table 2:** Risk of bias assessment of included studies. RoB 2, revised Cochrane Risk of Bias tool for randomized trials; SUMMIT, A Study of Tirzepatide in Participants With Heart Failure With Preserved Ejection Fraction and Obesity; BMI, body mass index; WHR, waist-height ratio; HF, heart failure; HFpEF, heart failure with preserved ejection fraction; 6MWD, six-minute walk distance.

Study	Risk of bias tool	Overall judgment	Key reason
Borlaug et al., 2025 [10]	RoB 2	Some concerns	Secondary analysis of SUMMIT; BMI/WHR subgroup and achieved weight-loss findings require cautious interpretation.
Packer et al., 2025 [11]	RoB 2	Low risk	Prespecified diabetes-stratified analysis within SUMMIT; diabetes was a randomization stratification variable.
Alghamdi et al., 2025 [12]	RoB 2	Some concerns	Pooled phase 2 trial analysis with mixed HF phenotypes; only 51% had HFpEF.
Borlaug et al., 2025 [13]	RoB 2	Some concerns	Prespecified secondary analysis; 6MWD subgrouping and weight-loss associations are partly exploratory.
Ferreira et al., 2025 [14]	RoB 2 for crossover trials	Some concerns	Open-label crossover design despite blinded endpoint assessment; short treatment periods.
Pandey et al., 2025 [15]	RoB 2	Some concerns	Prespecified pooled analysis, but frailty-stratified treatment effects are secondary subgroup findings.

Discussion

Principal Findings

Across the included randomized and trial-derived evidence, contemporary cardiometabolic therapies in HFpEF were associated with improvements across several clinically relevant domains, including heart failure events, patient-reported health status, exercise capacity, body weight, natriuretic peptides, adiposity measures, cardiac remodeling markers, and frailty burden. The established evidence from randomized trials supports the overall clinical efficacy of selected incretin-based and sodium-glucose cotransporter 2 (SGLT2) inhibitor-based therapies in appropriately selected HFpEF populations. Landmark trials such as EMPEROR-Preserved [16] and SUMMIT [17] have strengthened this evidence beyond ejection fraction-based classification by demonstrating therapeutic benefit in broad HFpEF and obesity-related HFpEF populations, respectively.

By contrast, the suggestion that diabetes status, adiposity distribution, functional limitation, frailty, renal profile, achieved weight loss, or ectopic fat modifies the magnitude or pattern of treatment response remains predominantly hypothesis-generating. These observations were derived mainly from secondary, pooled, subgroup, and stratification-based analyses and should not be interpreted as evidence that these characteristics can currently guide treatment selection. Rather, they identify potentially relevant domains for prospective evaluation and suggest that therapeutic response may not be fully captured by a single measure such as ejection fraction, diabetes status, or body weight change alone. Thus, the present findings support continued investigation of a phenotype-informed framework while recognizing that prospective phenotype-guided trials are required before such characteristics can be used as definitive treatment-selection tools.

Diabetes and Treatment Response

The findings of Packer et al. [11] provide an important clarification in the interpretation of diabetes as a modifier of treatment response in obesity-related HFpEF. Although patients with type 2 diabetes experienced less body-weight reduction with tirzepatide than those without diabetes, the benefits on cardiovascular death or worsening heart failure, Kansas City Cardiomyopathy Questionnaire Clinical Summary Score (KCCQ-CSS), 6MWD, quality-of-life measures, New York Heart Association (NYHA) functional class, paracardiac fat, and left ventricular mass were broadly similar across diabetes strata. Importantly, reduced weight loss in patients with diabetes did not correspond to an apparent reduction in cardiovascular or heart failure benefit. Thus, a smaller change in body weight should not, in isolation, be interpreted as evidence of diminished therapeutic efficacy. In diabetic HFpEF, body weight alone may therefore be an incomplete marker of therapeutic benefit, and treatment response may be better assessed through a broader set of outcomes, including symptoms, functional capacity, cardiac remodeling, adiposity distribution, and heart failure events [18]. These findings support a cautious but clinically useful interpretation: diabetes remains an important cardiometabolic phenotype in HFpEF, but it should not automatically be viewed as a marker of reduced therapeutic responsiveness.

Adiposity Phenotype

The findings of Borlaug et al. from the SUMMIT adiposity analysis support the view that obesity-related HFpEF should not be interpreted through BMI alone [10]. Although BMI remains useful as a simple marker of global adiposity, it does not distinguish lean mass from fat mass or characterize the anatomical distribution and metabolic activity of adipose tissue. Visceral adiposity is particularly relevant because it is more metabolically active than subcutaneous adiposity and may contribute disproportionately to systemic inflammation, insulin resistance, volume expansion, and cardiovascular dysfunction. Waist-height ratio can provide a practical surrogate measure of central or visceral adiposity, whereas imaging-derived epicardial and paracardiac fat measurements characterize local cardiac fat depots that are not captured by BMI. In the SUMMIT analysis, higher BMI was associated with greater symptom burden, physical limitation, volume expansion, and systemic inflammation, while waist-height ratio provided additional information about central adiposity, kidney dysfunction, and exercise impairment. The complementary findings of Alghamdi et al. further extend this concept by showing that dapagliflozin reduced epicardial adipose tissue and left ventricular mass in patients with type 2 diabetes and heart failure, despite the absence of parallel systemic inflammatory changes [12]. Together, these studies suggest that adiposity in HFpEF operates across several levels: global obesity reflected by BMI, central adiposity approximated by waist-height ratio, and local cardiac adiposity reflected by epicardial or paracardiac fat. This distinction is clinically important because weight loss may correlate with symptomatic and functional improvement, while visceral and local fat remodeling may provide additional mechanistic insight beyond changes in body weight alone.

Functional Phenotype

The semaglutide exercise-function analysis by Borlaug et al. highlights exercise limitation as a clinically meaningful treatment-response domain in obesity-related HFpEF [13]. Lower baseline 6MWD was associated with a broader adverse phenotype, including worse health status, higher BMI and waist circumference, greater systemic inflammation, higher N-terminal pro-B-type natriuretic peptide (NT-proBNP), and greater diuretic use. This suggests that impaired exercise capacity integrates multiple disease pathways rather than reflecting deconditioning alone. Semaglutide produced a placebo-adjusted improvement in 6MWD of 14.6 m at 20 weeks and 17.1 m at 52 weeks, alongside improvements in KCCQ-CSS, body weight, C-reactive protein, NT-proBNP, and the hierarchical composite outcome [13,19]. Proposed minimal clinically important differences for 6MWD in chronic heart failure generally range from approximately 15 to 35 m, although these thresholds remain uncertain and have not been firmly standardized for HFpEF [20]. The 52-week treatment effect therefore reached the lower end of this proposed range, whereas the 20-week effect was close to it. Accordingly, the mean improvement was statistically significant and potentially clinically meaningful but modest; patient-level responder analyses would provide a more definitive assessment of clinical relevance. These findings support the interpretation of 6MWD as more than a functional test; it may serve as a practical clinical readout of metabolic burden, congestion, inflammation, and cardiovascular reserve. Importantly, improvement in exercise capacity should not be reduced to weight loss alone, since functional recovery in HFpEF likely reflects several overlapping physiological changes relevant to patient-centered care.

Frailty as a Modifiable Phenotype

The frailty analysis by Pandey et al. adds an important clinical dimension to obesity-related HFpEF by suggesting that frailty should not be viewed only as a static risk marker or as a reason to anticipate limited treatment benefit [15]. In this pooled analysis of the STEP-HFpEF program, the greatest improvement in KCCQ-CSS with semaglutide was observed among the most frail participants, while weight loss was broadly similar across frailty strata. Semaglutide also reduced frailty burden at 52 weeks, suggesting that frailty in this population may reflect, at least in part, modifiable symptomatic, functional, metabolic, and inflammatory pathways rather than irreversible vulnerability alone. This finding is clinically relevant because frail patients with HFpEF are often perceived as less likely to benefit from weight-loss or incretin-based strategies. A more balanced interpretation is that frailty may identify patients with greater baseline symptom burden and greater potential for symptomatic improvement. However, frailty assessment is not yet sufficiently standardized for phenotype-guided treatment selection in routine HFpEF care. Available instruments-including deficit-accumulation indices, physical frailty phenotypes, and clinical frailty scales-measure partly different constructs and use different thresholds. The 34-variable cumulative deficit index used in this analysis may also be less practical for routine clinical implementation than shorter tools. Standardized instruments, validated cutoffs, and evidence that frailty-guided decisions improve outcomes are therefore needed before this phenotype can be routinely used to select therapy. Whether improvement in frailty burden translates into durable reductions in hospitalization, disability, institutionalization, or mortality also remains uncertain and requires prospective evaluation.

SGLT2-Based Cardiometabolic Optimization

The findings of Ferreira et al. and Alghamdi et al. support the interpretation of sodium-glucose cotransporter 2 (SGLT2) inhibition in HFpEF as a cardiometabolic, renal, and hemodynamic intervention rather than a glucose-lowering strategy alone [12,14]. In the SOGALDI-PEF trial, Ferreira et al. showed that dapagliflozin combined with spironolactone reduced NT-proBNP more than dapagliflozin alone and also improved blood pressure and albuminuria measures, suggesting potential additive biological effects in heart failure with mildly reduced ejection fraction (HFmrEF) and HFpEF [14]. However, this benefit was accompanied by greater estimated glomerular filtration rate (eGFR) decline, higher serum potassium, and more frequent hyperkalemia. These renal-electrolyte safety findings originated from a relatively small crossover trial involving 108 participants and short 12-week treatment periods; they should therefore be regarded as preliminary and should not be generalized to broader HFpEF populations without confirmation in larger, longer-duration trials. In parallel, Alghamdi et al. demonstrated that dapagliflozin reduced epicardial adipose tissue and left ventricular mass in patients with type 2 diabetes and heart failure, with changes occurring independently of BMI and without parallel systemic inflammatory improvement [12]. Together, these studies suggest that SGLT2-based therapy may influence HFpEF through several pathways, including natriuretic peptide reduction, blood pressure lowering, renal-hemodynamic effects, albuminuria reduction, local adiposity remodeling, and cardiac structural change. Clinically, this supports a framework in which SGLT2 inhibitors are interpreted not simply as antidiabetic agents but as foundational cardiometabolic therapies whose optimization requires attention to renal function, potassium, congestion, and structural remodeling [21].

Practical Phenotype-Guided Framework

The included evidence suggests a conceptual, but not yet prospectively validated, phenotype-guided framework for interpreting treatment response in HFpEF. Rather than treating diabetes, obesity, frailty, exercise limitation, ectopic fat, and renal function as isolated variables, these features may be understood as overlapping clinical signals that help characterize the dominant burden in an individual patient. Type 2 diabetes may indicate an altered metabolic response in which weight loss is less pronounced, but heart failure benefit may still be preserved, as shown by Packer et al. [11]. High BMI and central adiposity may reflect greater adiposity-driven HFpEF burden, while the findings of Borlaug et al. [13] suggest that reductions in weight and waist measures can track with improvements in symptoms and exercise capacity. Low 6MWD may identify patients in whom functional limitation reflects the combined effects of adiposity, congestion, inflammation, and impaired physiological reserve. Frailty, as shown by Pandey et al. [15], may not simply mark vulnerability but may also identify patients with substantial symptomatic burden and potential for improvement. Epicardial or paracardiac fat, supported by the findings of Alghamdi et al. [12], may represent a local cardiometabolic remodeling signal, while renal function and potassium profile define the safety boundary for combination strategies such as SGLT2 inhibition with mineralocorticoid receptor antagonism, as shown by Ferreira et al. [14]. This proposed framework is intended only to organize and interpret the emerging evidence. It has not been prospectively tested as a treatment algorithm and should not currently replace guideline-directed treatment selection or established clinical risk assessment.

Limitations and Future Directions

Several limitations should guide the interpretation of this evidence. Much of the included data came from secondary, prespecified, pooled, or subgroup analyses rather than trials originally designed to test phenotype-guided treatment selection. Several publications were also derived from overlapping parent-trial populations. Specifically, two analyses originated from the SUMMIT population [10,11], while two originated from the pooled STEP-HFpEF and STEP-HFpEF DM populations [13,15]. Consequently, the six included publications represent fewer fully independent participant cohorts, which limits the diversity and breadth of the evidence base and may give disproportionate weight to findings from these trial programs. The study by Alghamdi et al. [12] included mixed heart failure phenotypes, with only about half of the cohort classified as HFpEF; its findings are therefore best interpreted as mechanistic support rather than definitive HFpEF-specific clinical evidence. In addition, many extracted outcomes involved symptoms, health status, exercise function, biomarkers, adiposity measures, frailty burden, or cardiac remodeling rather than hard mortality endpoints. Phenotypes such as waist-height ratio, epicardial adipose tissue, paracardiac fat, frailty indices, and 6MWD are clinically informative but are not yet standardized as treatment-selection tools in routine HFpEF care. A formal Grading of Recommendations Assessment, Development and Evaluation (GRADE) assessment was not performed. Therefore, the certainty of evidence across outcomes was not systematically rated, which limits the strength of conclusions that can be drawn from the synthesis. Future studies should prospectively test phenotype-guided treatment algorithms integrating diabetes status, adiposity distribution, frailty, exercise capacity, renal profile, and imaging-based fat depots to determine whether these markers improve treatment selection beyond ejection fraction, BMI, and conventional clinical risk assessment.

## Conclusions

Recent randomized and trial-derived evidence suggests that cardiometabolic and functional characteristics may provide additional context for interpreting treatment response in HFpEF beyond diabetes status, BMI, or ejection fraction alone. Across the included studies, incretin-based and sodium-glucose cotransporter 2 inhibitor-based therapies were associated with improvements in heart failure outcomes, symptoms, exercise capacity, adiposity measures, natriuretic peptides, cardiac remodeling markers, and frailty burden. However, evidence regarding differences in response across specific phenotypes was derived predominantly from secondary, pooled, subgroup, and exploratory analyses, including publications with overlapping trial populations. Reduced weight loss in patients with diabetes did not necessarily correspond to reduced heart failure benefit, while symptoms, functional capacity, ectopic fat, renal-hemodynamic markers, and frailty may provide complementary measures of therapeutic response. Nevertheless, these observations should be considered hypothesis-generating and do not currently establish validated criteria for phenotype-guided treatment selection. Prospective trials are needed to determine whether integrating diabetes status, adiposity distribution, frailty, exercise limitation, renal safety, and imaging-based adiposity markers improves treatment selection and clinical outcomes beyond established HFpEF management approaches.
